# Epigenetic Biomarkers Screening of Non-Coding RNA and DNA Methylation Based on Peripheral Blood Monocytes in Smokers

**DOI:** 10.3389/fgene.2022.766553

**Published:** 2022-02-11

**Authors:** Xiaowei Huang, Bian Wu, Fangxue Zhang, Fancheng Chen, Yong Zhang, Huizhi Guo, Hongtao Zhang

**Affiliations:** ^1^ Department of Orthopedics, The First Affiliated Hospital of Soochow University, Suzhou, China; ^2^ National Clinical Research Center of Kidney Diseases, Jinling Hospital, Nanjing University School of Medicine, Nanjing, China; ^3^ Knee Surgery Department of the Institute of Sports Medicine, Beijing Key Laboratory of Sports Injuries, Peking University Third Hospital, Peking University, Beijing, China; ^4^ Department of Orthopaedics, Zhongshan Hospital, Fudan University, Shanghai, China; ^5^ The First Institute of Clinical Medicine, Guangzhou University of Chinese Medicine, Guangzhou, China

**Keywords:** epigenetics, DNA methylation, non-coding RNA, bioinformatics, cigarette smoking

## Abstract

This study aims to use bioinformatics methods to determine the epigenetic changes in microRNA expression and DNA methylation caused by cigarette smoking. The data of mRNA, miRNA expression, and methylation microarray were obtained from the GEO database to filter differentially expressed genes (DEGs), differentially expressed miRNAs (DEMs), and methylated CpG probes (DMPs) through the limma package. The R clusterProfile package was used for functional annotation and enrichment analysis. The protein-protein interaction (PPI) network was constructed by the String database and visualized in Cytoscape software. Starbase database was employed to predict lncRNA and CirRNA based on the sequence of miRNA, and to establish a regulatory network of ceRNA. By overlapping DEG and DEM, 107 down-miRNA-targeted up-regulated genes and 65 up-miRNA-target down-regulated genes were obtained, which were mainly enriched in autophagy signaling pathways and protein ubiquitination pathways, respectively. In addition, 324 genes with low methylation and high expression and 204 genes with high methylation and low expression were respectively related to the degeneration of the nervous system and the function of the cardiovascular system. Interestingly, 43 genes were up-regulated under the dual regulation of reduced miRNA and hypomethylation, while 14 genes were down-regulated under the dual regulation of increased miRNA and hypermethylation. Ten chemicals have been identified as putative therapeutic agents for pathological conditions caused by smoking. In addition, among these genes, HSPA4, GRB2, PRKCA, and BCL2L1 could play a fundamental role in related diseases caused by smoking and may be used as the biomarkers for precise diagnosis and targets for future therapies of smoking-related diseases.

## Introduction

Smoking can cause a variety of tumors and non-tumor diseases. It is estimated that 40% of human cancers are smoking-related which are responsible for more than 5 million preventable deaths worldwide per year (Ambrose and Barua, 2004). Many chemicals in tobacco are toxic. It has been reported that there are more than 69 harmful components, including 10 kinds of polycyclic aromatic hydrocarbons, 10 kinds of nitrosamines, eight kinds of heterocyclic amines, and six kinds of heterocyclic hydrocarbons generated when smoking (Benowitz, 2003). Smoking can impair natural killer cell function in the innate immune system, thereby weakening the function of tumor surveillance and inhibiting atypical cell growth (Yasuma et al., 2021). Like most environmental exposures, it does not directly cause certain diseases. On the contrary, it can increase the susceptibility to chronic diseases, such as coronary atherosclerosis and metabolic disorders. The pathogenesis caused by cigarette smoking includes inflammation and immune suppression, reactive oxidative species generation, and apoptosis (Yoon et al., 2021). However, the exact mechanism leading to smoking-related diseases and deaths remains largely unknown. Recently, epigenetic regulation has become the focus of research, which can effectively explain many genomic effects that cannot be explained by protein-coding genes (Nakamura et al., 2021; J. L.; Zhang et al., 2019).

MicroRNA is a single-stranded RNA with no protein-coding function, which regulates gene expression after transcription in numerous pathogenic signal transduction cascades with very little variation among species (Xu et al., 2017). Schembri et al. recently identified 28 miRNAs in smokers. Compared with non-smokers, these miRNAs are differentially expressed in the tracheal epithelium of smokers. Most of these miRNAs (82%) are down-regulated (Zhang et al., 2019). The changes in miRNA expression caused by smoking are thought to regulate the inflammation, generation of reactive oxidative species, xenobiotic metabolism, and carcinogenesis (Schembri et al., 2009). A recent study further showed that smoking caused 126 miRNAs in lung tissue to be down-regulated by at least 2 times, and 24 miRNAs were down-regulated by more than 3 times. These miRNA expression changes are related to the up-regulation of 107 genes, which regulate the expression of protein-coding genes related to stress, cell cycle regulation, and vascular formation (Willinger et al., 2017; Xu et al., 2018).

Recently, it was found that alterations of DNA methylation could be possible pathogenesis that mediate CS-induced diseases (Buck et al., 2021). It has been reported that, for current smokers, differential CpG island methylation occurred in almost one-third of human transcriptome (Bakulski et al., 2019). In addition, maternal smoking exposure has a great impact on the DNA methylation of infants. Suter et al. compared 36 matched placental samples with gene expression arrays and methylation arrays. 438 deferentially expressed genes showed a significant correlation with aberrant methylation. Several of them belong to characteristic pathways of oxidative phosphorylation, which indicated smokers have increased oxidative damage markers. These CpGs sites can be used as potential biomarkers to predict smoking-related disorders (Andersen et al., 2021). DNA methylation is mainly catalyzed by DNA methyltransferase (DNMT). Cigarette smoking can up-regulate DNMT1 expression, thus promoting the methylation process. The researchers revealed that compared with non-smokers, the expression of DNMT1 in the lung tissue of smokers was significantly higher (Lai et al., 2017).

So far, innumerable studies have reported abnormal DNA methylation and aberrant miRNA expression between smokers and non-smoking controls (Nozoe et al., 2002; Stroud et al., 2016; Sun et al., 2015). However, a comprehensive regulatory network on smoking-related microRNA and DNA methylation changes has not been established yet. Therefore, a thorough analysis of the regulatory network is needed to depict the correlation between genetic and epigenetic changes. In this study, the mRNA expression profile microarray data, miRNA expression microarray, and DNA methylation microarray were systematically analyzed to determine the core genes and pathways that lead to smoking-related damage through epigenetic regulation.

## Methods

### Data Retrieval and Annotation

Expression profile microarrays (GSE87072), non-coding RNA microarray (GSE69960), and gene methylation profile data sets (GSE53045) were retrieved and analyzed. GSE87072 (platform: GPL570 Affymetrix Human Genome U133 Plus 2.0 Array) included peripheral blood mononuclear cells (PBMC) samples from 40 healthy male smokers and 40 healthy non-smokers as controls. The GSE69960 data set included peripheral blood samples of 28 smokers and 12 non-smokers. GPL18402 Agilent-046064 Unrestricted _Human_ miRNA_ V19.0_ Microarray was used for measurement and annotation. In GSE53045, 111 PBMC samples were analyzed for genome-wide DNA methylation, of which 50 were smokers and 61 were non-smokers. Illumina HumanMethylation450 BeadChip was used for analysis, and the annotation platform file was GPL13534.

### Data Processing

The matrix files of the above three data sets were downloaded and the R language limma package was utilized to screen out deferentially expressed genes (DEGs), miRNAs (DEMs), and methylated CpG probes (DMPs) (W. Zhu W. et al., 2020). Subsequently, DEGs, DEMs, and DMPs were filtered out, using *p* <0.05 and abs (t) > 2 as cutoff values. DMPs located in the gene region were well-anchored to the corresponding genes and defined as differentially methylated genes (DMGs). The single methylation value is merged into each gene promoter represented in the HM4^50^K platform, using the median of the CpG probe methylation value to locate in the promoter region such as 1500 nucleotides from the transcription start site TSS1500, 200 nucleotides from the transcription start site TSS200, 5'UTR and the first exon (Teschendorff et al., 2013). In addition, the CpG probes in the sex chromosomes were removed for data extrapolation across gender. To find out the worth-noting epigenetic changes in smokers, Venn diagrams were used to take the intersection of DEGs, target genes predicted by DEMs, and DMPs anchored genes (DMGs) to identify overlapping genes.

### Target Gene Prediction Based on miRNA Sequence and miRNA-Transcription Network Construction

Micro-RNA list was imported to the miRWalk 3.0 online database to predict target genes, which comprises three sub-databases, namely, miRDB, Targetscan, and miRTarBase. Among them, miRTarBase was the unique database, of which all the data have been experimentally validated (Fan et al., 2021). As a result, the genes predicted by miRTarBase w/o other two databases were considered to be the target genes of DEM. To align DEM and DEGs, Cytoscape software (version 3.8.2) was utilized to illustrate the entire miRNA-mRNA regulation network (Chen et al., 2021).

### Prediction of lncRNA and Cir-RNA and Construction of the ce-RNA Regulation Network

Micro-RNAs were input into StarBase (v 3.0) for the prediction of lncRNA and circRNA according to the sequencing targeting (Li et al., 2014; Yang et al., 2011). The filtration parameters were set as low stringency (≥1) in terms of CLIP data and degradome data. The top 5 predicted lncRNA and circRNA were chosen, based on which the ceRNA regulation networks were visualized using Cytoscape.

### Functional Annotation and Pathway Enrichment Analysis

GO and KEGG enrichment analysis was conducted to analyze the function and involved biological process of the overlapped genes of DEG and other data sets. Funrich software and R package clusterPorfiler was employed to complete the enrichment analysis which was then visualized via R package ggplot2 (Ito and Murphy, 2013; Maag, 2018). In addition, the Cytoscape plug-in ClueGO was used to analyze the relationship between the enriched biological processes.

### Topological Analysis of Protein Function and Establishment of Interaction Network

Protein-coding gene lists of hypo-methylated up-expressed and hyper-methylated down-expressed genes were input into the STRING database to analyze the interaction network of encoding proteins. The output tsv files were then imported to Cytoscape for protein-protein interaction (PPI) network visualization, and the molecular complex detection (MCODE) plugin was utilized to screen out modules within the PPI network (Chen and Chiang, 2021; Osterman et al., 2020).

### Hub Gene Distribution Among Different Tissues

In order to provide a deeper insight into the up-regulation and down-regulation of gene expression across the human body, up-regulated and down-regulated hub gene lists were imported to the MERAV online database (Shaul et al., 2016) and heat maps of hub genes were then plotted to illustrate deferential expression among different tissues.

### Therapeutic Strategy Prediction

The CMap database is an open access online tool that predicts potential drugs and has been used in this study to screen out potential compounds that can bind to the proteins (Gillbro et al., 2015; Khater et al., 2015), of which the expression was altered by cigarette smoking. The match possibility between the chemical substance and the gene inquired was evaluated by connection score ranging from −1 to 1 and *p* < 0.05 was set as the cutoff value.

### Molecular Docking Analysis

Maestro (version 10.2) was used to more intuitively display and predict the interaction between the compound and the target protein encoded by hub genes (Shah et al., 2020). The protein crystal structures were obtained from the Protein DataBank database (Pascarella et al., 1996) and the chemical structures were obtained from the PubChem online database(Kim, 2021; Zaslavsky et al., 2021). The protein and chemical structures were imported into the Maestro software. After the assignment of bond orders, the addition of hydrogens, creation of zero-order bonds to metals, and creation of disulfide bonds, the preparation is completed and the structures are ready for docking.

## Results

### Data Description and Probe Screening as Well as Annotation

**FIGURE 1:**
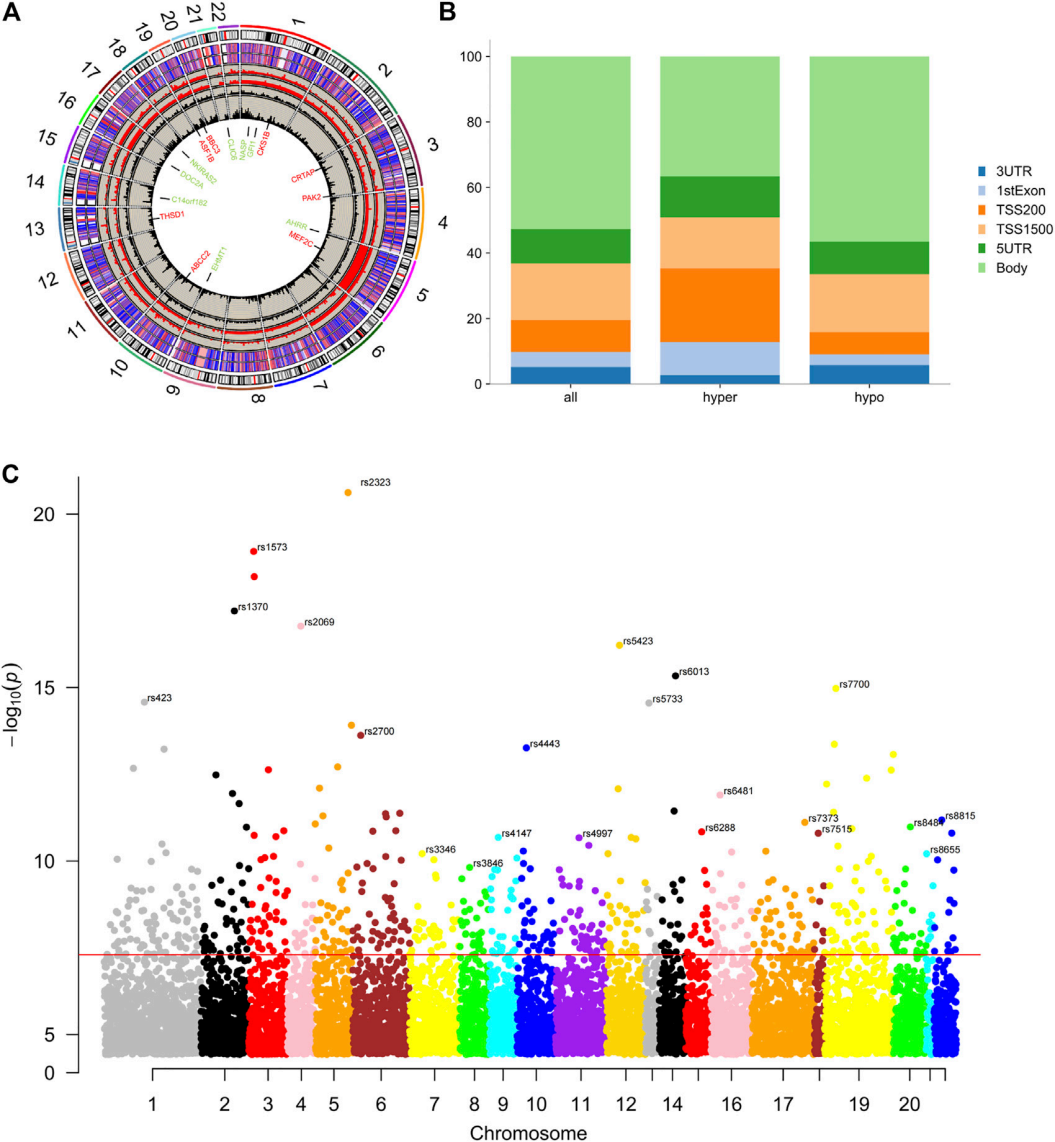
Differential DNA methylation distribution. **(A)**, Circus plot of CpGs. Autosomal chromosomes are shown in a clockwise direction from 1 to 22 in the outermost circle. Chromosomes X, Y were excluded from the analysis. Red and green-labeled genes correspond to the top 8 hypermethylated and hypomethylated genes, respectively. The two innermost circles represent the frequency of the filtered hypermethylated (inner) and hypomethylated (outer) CpG islets. The two middle circles demonstrate the histogram of *p* values of hypermethylated (inner) and hypomethylated (outer) CpG islets. The two outermost circles show the heat maps with two different sample sets of methylated CpG islets. **(B)**, Bar plot of differentially methylated CpGs throughout each genomic region. The body methylated CpG islets were excluded for further analysis. **(C)**, Manhattan plot of epigenome-wide association results showing −log_10_ (*p*-value) labeled with the red line.

**FIGURE 2:**
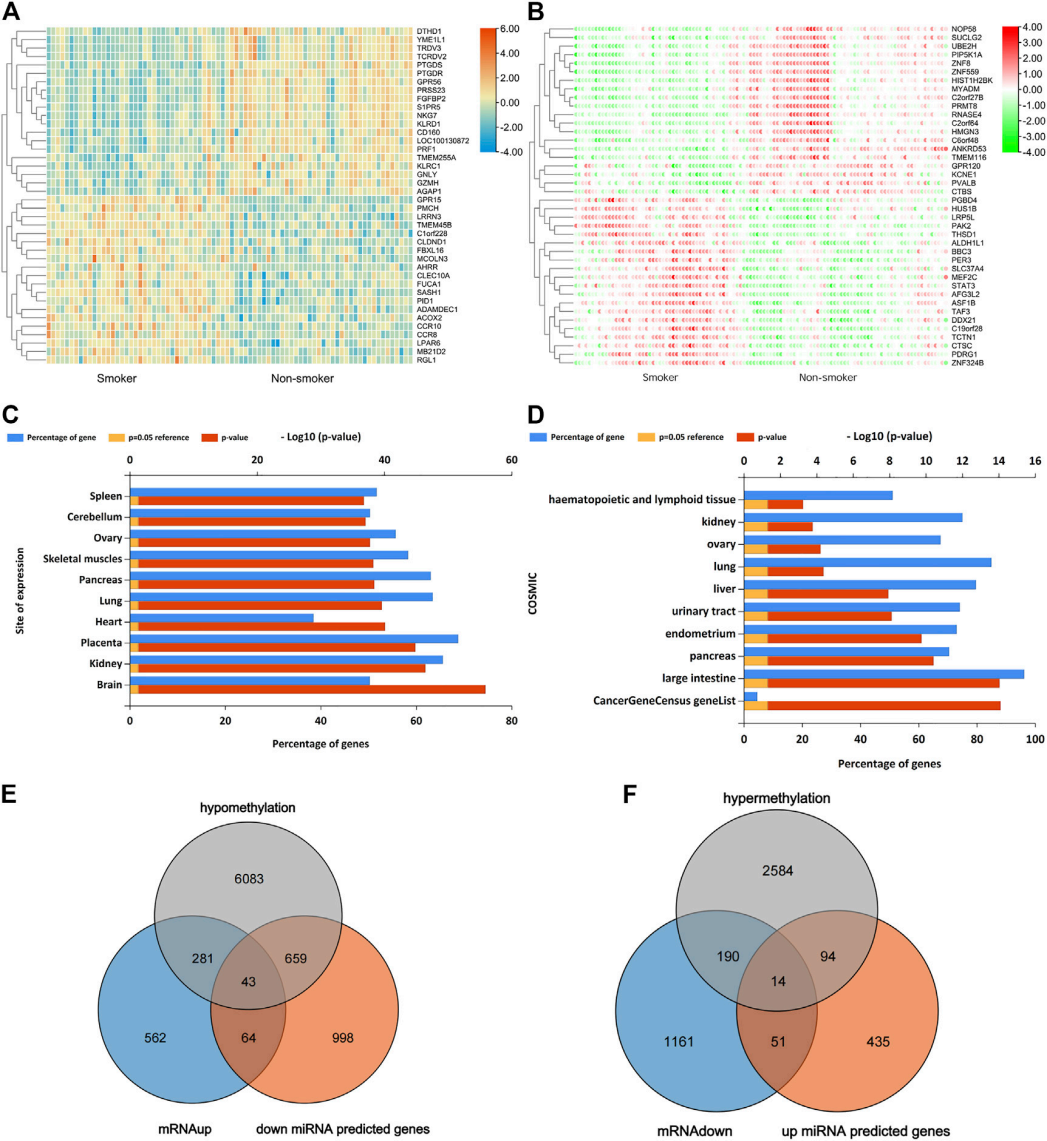
**(A)**, Top 40 DEGs (20 up-regulated genes and 20 down-regulated genes) of GSE87072. **(B)**, Top 40 DMGs (20 hypermethylation genes and 20 hypomethylation genes) of GSE53045. Red indicates that the expression of genes is relatively up-regulated, blue or green indicated that the expression of genes is relatively down-regulated. **(C)**, Enrichment analysis of DEMs expressed in different sites. **(D)**, Functional enrichment analysis of DEMs based on the COSMIC database. **(E and F)**, Venn graph for all the overlapped genes including up-regulated 43 genes and 14 down-regulated genes, respectively.

In GSE87072, 2366 mRNAs were identified from smokers’ PBMC samples, including 950 up-regulated as well as 1416 down-regulated DEGs. In the meanwhile, 10 DEMs with enhanced expression and 67 DEMs with reduced expression were picked in the miRNAs data set GSE69960. As for the gene methylation microarray GSE53045, 11,611 hypomethylated CpG sites in 7066 genes and 3360 hypermethylated CpG sites in 2882 genes have been found. The CpG probes located on the XY chromosome and the body of the gene sequence were excluded for further analysis. The distribution of methylated CpG sites located on autosomes was demonstrated on the circus plot in Figure 1A. Bar plot of Figure 1B showed the proportion of six genomic subregions where methylated CpG islets were located. In addition, the Manhattan plot indicated that differentially methylated genes (DMG) anchored by methylated CpG islets were scattered on autosomes in an evenly distributed manner, as illustrated in Figure 1C. Through the approach of overlapping DEG and DEM predicted target genes, 107 up-regulating genes, and 65 down-regulating genes were obtained. Figures 2A,B showed the heat-maps of the first 40 DEGs and DMGs (20 enhanced expressed genes and 20 reduced expressed genes in each heat-map), which were deferentially expressed in the smoker and non-smoker control groups. The Funrich database was utilized for miRNA enrichment analysis to explore tissue expression distribution of DEMs, as shown in Figures 2C,D.

### The Overlap of Up-Regulated Genes and Target Genes Predicted by Lowly-Expressed miRNAs

**FIGURE 3:**
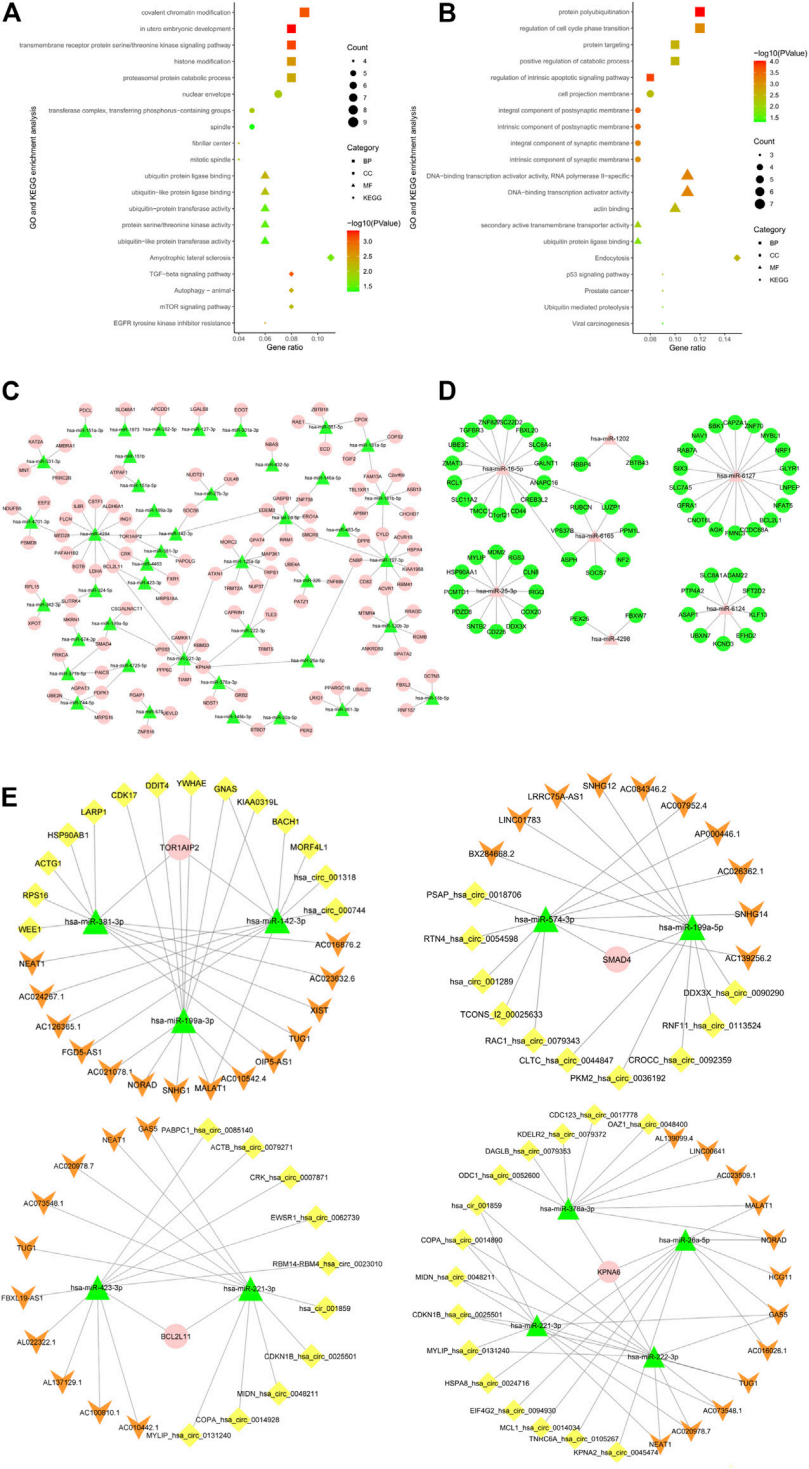
The visualized regulatory network and enrichment bubble graph for miRNA-targeting DEGs. **(A)**, KEGG and GO enrichment bubble graph for overlapped up-regulated genes. **(B)**, KEGG and GO enrichment bubble graph for overlapped down-regulated genes. **(C)**, Regulatory network graph of 43 low-expression miRNAs. **(D)**, Regulatory network graph of seven high-expression miRNAs. **(E)**, ceRNA regulatory networks of TOR1AIP2, KPNA6, BCL2L11 and SMAD4.

Venn diagram demonstrated 107 overlapped up-regulated genes that can be targeted by lowly-expressed miRNA, as shown in Figure 2E. Functional enrichment analysis determined that the top 5 gene ontology terms in three aspects (cell component, molecular function, and physiologic process) which met the threshold of statistical significance were mainly related to the chromatin modification, transferase activity, and ubiquitin-protein ligase binding, as shown in Figure 3A. KEGG pathway enrichment indicated that they may play an important part in amyotrophic lateral sclerosis, TGF-beta signaling pathway, autophagy, mTOR signaling pathway, and EGFR tyrosine kinase inhibitor resistance. To further depict the explicit miRNA-mRNA regulation panorama in the smoking-related pathogenesis, a miRNA regulation network was constructed in Figure 3C. KPNA6 and TOR1AIP2 were targeted by four miRNAs. SMAD4, BCL2L11 were targeted by three miRNAs. MED28, SLITRK4, LDHA, PAICS, PDPK1, BTBD7, VPS53, CRK, ATPAF1, SOCS6, CPOX, FAMBA, CYLD, SMCR8, ACVR1, GPAT4, and TRPS1 were regulated by two miRNAs. The left genes like PDCL, SLC48A1 and APCDD1 were regulated by one miRNA. In the present study, an online database Starbase 3.0 was employed to predict the targeted ncRNA and to establish the ceRNA regulatory network of the BCL2L11, KPNA6, TOR1AIP2, and SMAD4, as shown in Figure 3E.

### The Overlap of Down-Regulated Genes and High-Expressed miRNAs Predicted Target Genes

Venn diagram demonstrated 65 overlapped down-regulated genes that can be targeted by high-expressed miRNA, as shown in Figure 2F. The enrichment analysis demonstrated that the GO terms were mainly related to protein polyubiquitination, cell cycle, catabolic process, transcription activity, and apoptosis. Enriched KEGG analysis items are endocytosis, p53 signaling pathway, prostate cancer, ubiquitin-mediated proteolysis, and viral carcinogenesis, as shown in Figure 3B. As illustrated by the miRNA-mRNA network, as plotted in Figure 3D, two genes ASPH and PPM1L can be regulated by two miRNAs in Figure 3B. For hsa-miR-451a, hsa-miR-6076, and hsa-miR-486-5p, target genes predicted by miRWalk were not found in DEGs. As a result, they were not included in the network.

### Highly Expressed and Hypomethylated Genes

**FIGURE 4:**
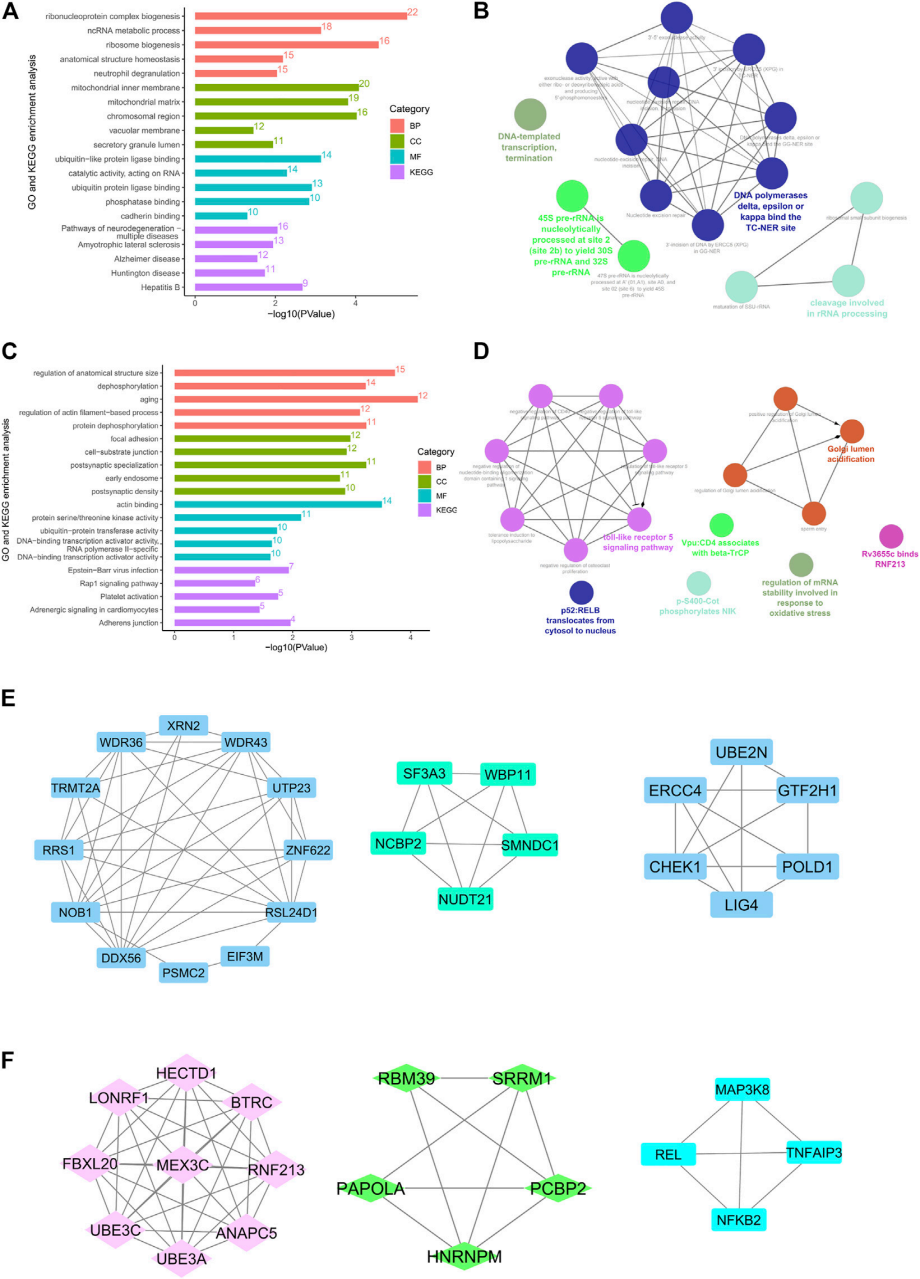
The protein-protein interaction (PPI) network and enrichment bar graph for methylation-related DEGs. **(A and C)**, GO and KEGG enrichment bar graph for hypomethylation–up-regulated genes **(A)** and hypermethylation–down-regulated genes **(C)**. **(B and D)**, Reactome and KEGG pathway enrichment visualized by CluoGO of hypomethylation and high-expression hub genes **(B)** and hypermethylation and low-expression hub genes **(D)**. **(E and F)**, Top three modules PPI network of hypomethylation–high-expression genes **(E)** and hypermethylation–low-expression genes **(F)**.

Enrichment analysis was performed on 324 hypomethylated and highly expressed genes. With the threshold value of *p*-value < 0.05, the enriched genes were related to ribonucleoprotein complex biogenesis, ncRNA metabolic process, ribosome biogenesis, ubiquitin−like protein ligase binding. It was showed that pathways were enriched in neurodegeneration, Amyotrophic lateral sclerosis, Alzheimer’s disease, Huntington disease, and Hepatitis B, as shown in Figure 4A. Proteins interaction analysis was constructed with the String database. A total of 318 nodes and 670 edges were plotted in the PPI network. The top ten genes sorted by degree of connectivity were regarded as hub genes, including UBE2N, HSPA4, HSPA9, HSPD1, EEF2, ATP5A1, CCT4, PSMC2, CCT8and MRPL4. Among these 10 central genes, UBE2N has the highest degree (degree = 28). In addition, in Figure 4E, the MCODE plug-in identified the top three important modules with scores of 7.1, 5, and 4.8, and functional annotations were analyzed and visualized using the ClueGO plug-in from Cytoscape. The biological process and Reactome pathway analysis showed that Module 1-3 were related to DNA-templated transcription termination, DNA polymerases binding and rRNA processing, as shown in Figure 4B.

### Lowly Expressed and Hypermethylated Genes

Enrichment analysis was performed on 204 hypermethylated and lowly expressed genes. The top 15 GO items were enriched with the DAVID database and a bubble plot was presented using the ggplot2 package (Figure 4C). KEGG analysis demonstrated enriched pathways for Epstein−Barr virus infection, Rap1 signaling pathway, Platelet activation, Adrenergic signaling in cardiomyocytes, and Adherens junction. Proteins interaction analysis was constructed with the String database. A total of 203 nodes and 193 edges were displayed in the PPI network. The top ten genes sorted by degree of connectivity were regarded as hub genes, including BTRC, UBE3A, UBE3C, ANAPC5, BCL2L1, FBXL20, RNF213, HECTD1, MEX3C, and LONRF1. Among these 10 hub genes, BTRC got the highest degree (degree = 14). After analysis of the MCODE plug-in, the three important modules with scores of 9.0, 4.5, and 4.0 were selected (Figure 4F). The biology process, KEGG, and Reactome pathway enrichment analysis of modules 1-3 showed enrichment in toll-like receptor 5 signaling pathway, Golgi lumen acidification, regulation of mRNA stability involved in response to oxidative stress, and p52: RELB translocation from cytosol to nucleus, as shown in Figure 4D.

### DEGs Regulated by Both miRNA and DNA Methylation

**FIGURE 5:**
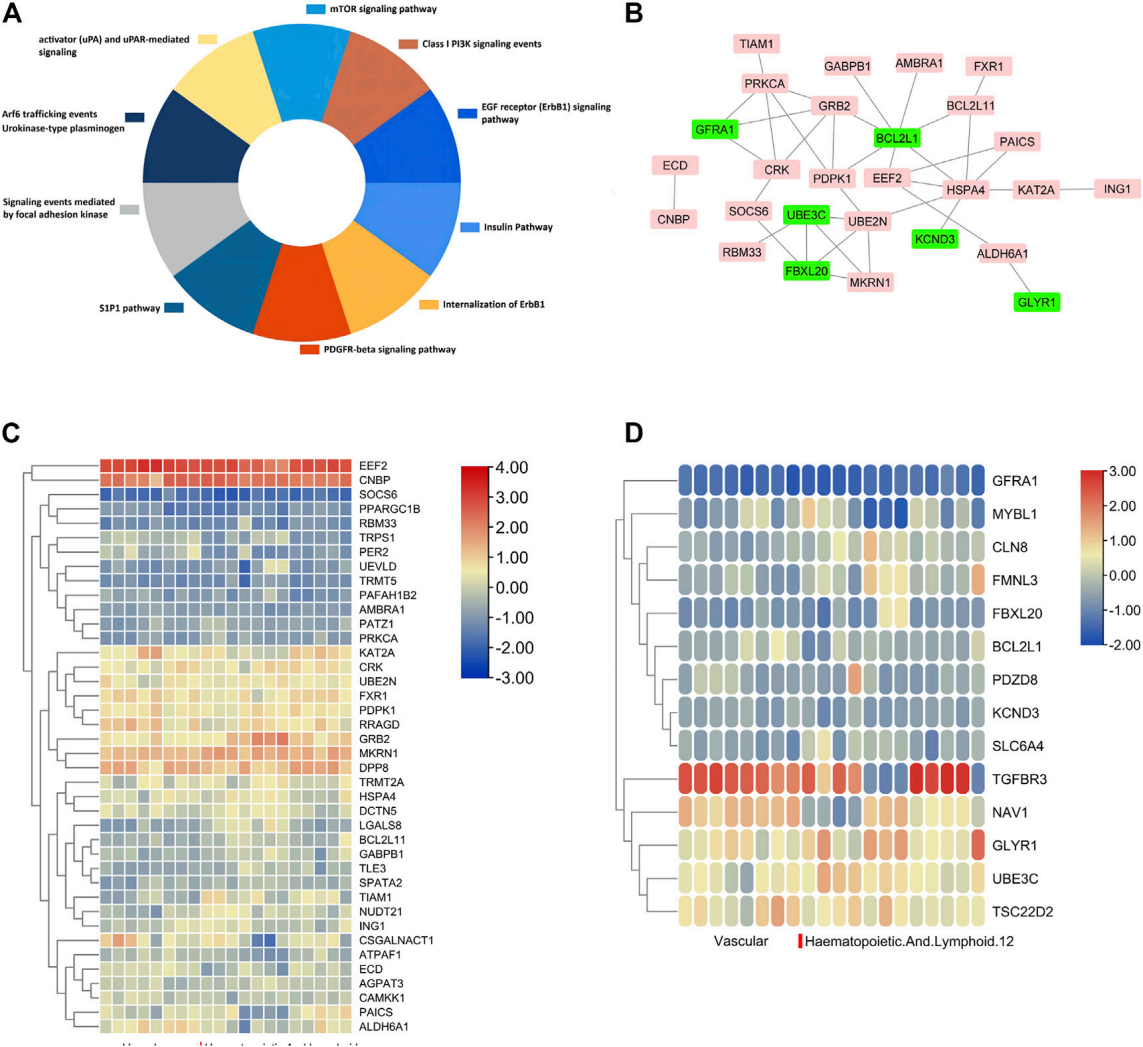
Details for all the overlapped genes. **(A)**, KEGG and biological process enrichment analysis of dual regulated genes, respectively. **(B)**, PPI network of the overlapped dual regulated genes including an up-regulated label with red color and down-regulated genes with green color. **(C and D)**, Heatmap of 43 up-regulated **(C)** and 14 down-regulated genes **(D)** expressed in different tissues.

It was worth noting that some DEGs were simultaneously regulated by DNA methylation and miRNA binding, indicating important and intricate regulation behind smoking. GRB2, UBE2N, TRMT2A, AMBRA1, CAMKK1, ZNF516, and other 37 genes were up-regulated with hypomethylation and low miRNA expression (Figure 5C). At the same time, 14 genes including FBXL20, CLN8, PDZD8, and NAV1 were down-regulated during hypermethylation and high miRNA expression (Figure 5D). Supplementary Table S4 summarizes DNA methylation sites and their relationship with CpG islands. In addition, the results of the KEGG pathway enrichment analysis were shown in Figure 5A. In order to provide a deeper insight into the different patterns of gene expression among different tissues, up-regulated and down-regulated hub gene lists were imported to the MERAV online database, and heat maps of hub genes were then plotted, as shown in Figures 5C,D, to illustrate deferential expression in hematopoietic system and vasculature. In the group of 43 up-regulated genes, EEF2, CNBP, MKRN1, and DPP8 demonstrated significant high expression in the hematopoietic system and vasculature, while the expression of SOCS6 and PPARGC1B genes were down-regulated significantly. Among the 14 down-regulated genes, TGFBR3 was significantly up-regulated in the hematopoietic system and vasculature, while GFRA1 and MYBL1 were significantly down-regulated.

**FIGURE 6:**
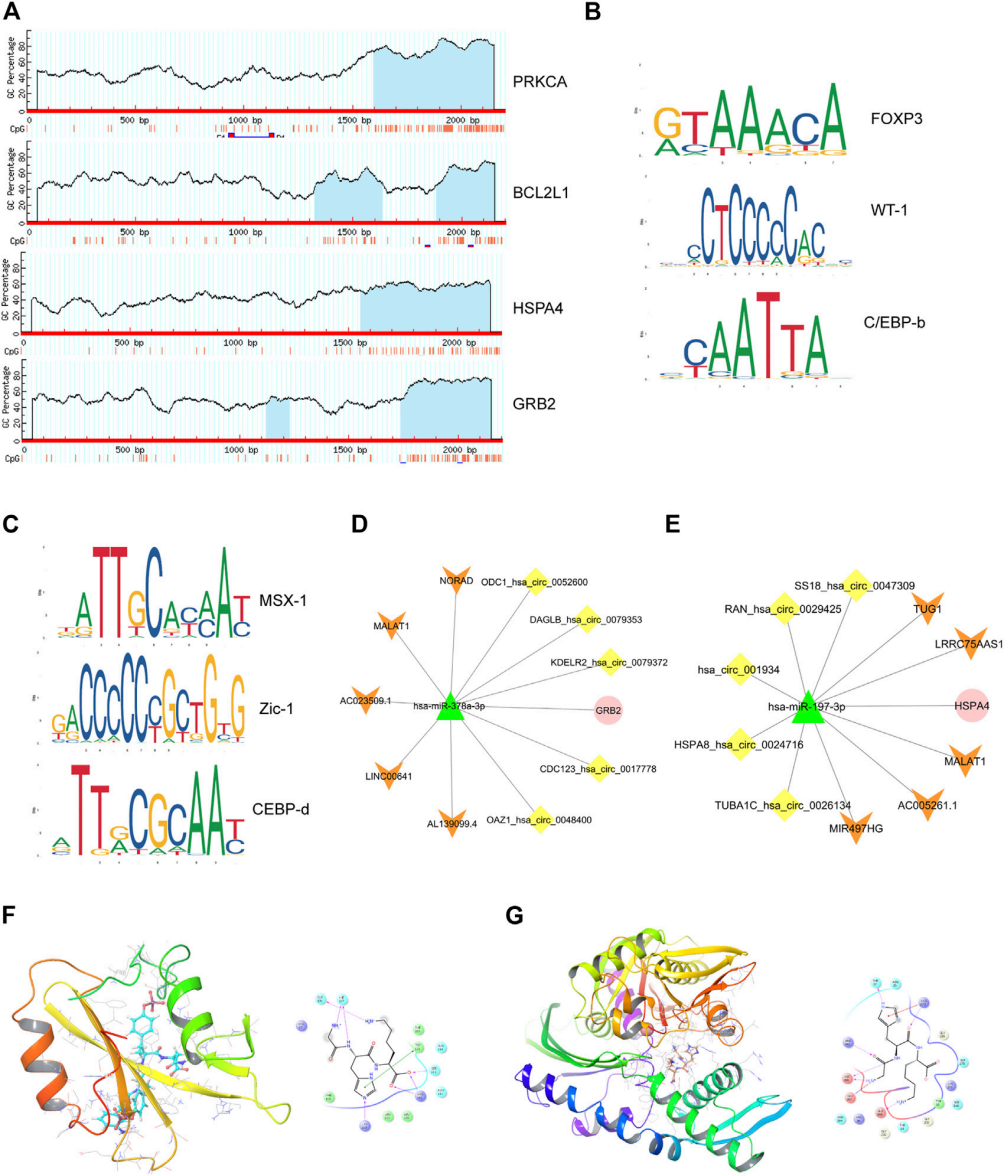
Comprehensive epigenetic regulation prediction of the four hub genes. **(A)**, CpG islands prediction analysis of 4 hub genes. **(B)**, Representative predicted transcription factors of target GRB2 islands enriched in DMRs. **(C)**, Representative predicted transcription factors of target HSPA4 islands enriched in DMRs. **(D and E)**, CeRNA regulatory network of GRB2 and HSPA4. **(F)**, simulation of molecular docking pattern between GRB2 (PDB ID: 6ICG) and Gly-His-Lys. **(G)**, simulation of molecular docking pattern between HSPA4(PDB ID: 3QFP) and Gly-His-Lys.

A gene list of 43 up-regulated and 14 down-regulated dual-regulation genes were uploaded to the CMap website to speculate potential compounds to alleviate or reverse the pathogenesis caused by smoking. According to *p*-value, 4 chemicals with the smallest *p* values were chosen as potential therapeutic chemicals against smoke-induced pathological changes, as listed in Table 1. In addition, protein-protein interaction (PPI) analysis was conducted on 57 abnormally expressed genes with overlapping miRNA and methylation regulation, as shown in Figure 5B. The four hub genes from PPI with top connectivity scores were further screened and analyzed, including three up-regulated genes (HSPA4, GRB2, PRKCA) under hypomethylation plus low miRNA and one down-regulated gene BCL2L1 with hypermethylation as well as high miRNA. Figure 6A illustrated the CpG island prediction analysis on the 2200 pb sequence downstream of the promoters in 4 genes. There are two predicted CpG islands in BCL2L1 and GRB2 promoter regions respectively. There is one predicted CpG island in HSPA4 and PRKCA promoter regions respectively. PROMO, a virtual laboratory for the identification of putative transcription factor binding sites (TFBS) in DNA sequences was employed to determine the three representative binding sites of promoters in the GRB2 and HSPA4 promoter regions (Figures 6B,C). The ceRNA network of GRB2 and HSPA4 has been predicted to understand epigenetic regulation from a more comprehensive perspective, as shown in Figures 6D,E. In addition, to determine how the predicted chemical substance directly binds to the hub-gene-encoding proteins, the binding patterns were simulated as shown in Figures 6F,G.

**TABLE 1:** Top ten chemicals were predicted as putative therapeutic agents for smoking-related conditions.

cmap name	Pubchem ID	Molecular formula	Mean	n	Enrichment	*p*
Gly-His-Lys	73587	C_14_H_24_N_6_O_4_	0.631	3	0.947	0.00018
Wortmannin	312145	C_23_H_24_O_8_	0.276	18	0.484	0.00022
Tetracaine	5411	C_15_H_24_N_2_O_2_	0.634	3	0.925	0.00082
Diethylstilbestrol	448537	C_18_H_20_O_2_	0.245	6	0.72	0.00133
Calcium pantothenate	443753	C_18_H_32_CaN_2_O_10_	−0.513	4	−0.823	0.00189
Pivampicillin	33478	C_22_H_29_N_3_O_6_S	−0.551	4	−0.812	0.00229
Nadolol	39147	C_17_H_27_NO_4_	0.363	4	0.806	0.00265
Guanadrel	38521	C_10_H_19_N_3_O_2_	0.439	5	0.74	0.0027
Thapsigargin	446378	C_34_H_50_O_12_	0.55	3	0.885	0.00312
Monastrol	2987927	C_14_H_16_N_2_O_3_S	0.363	8	0.582	0.00405

## Discussion

Promoter sequence methylation and changes in miRNA expression play an important role in smoking-induced diseases by altering gene transcription activities (Lamadrid-Romero et al., 2018). As a result, DNA methylation patterns and particular miRNAs can be regarded as instrumental biomarkers to evaluate smoking-related pathological conditions (L. Wang et al., 2018). In the current study, the data of miRNA microarray, DNA methylation microarray, and mRNA microarray were systematically analyzed, as shown in Figures 1, 2, and the differential analysis between the smoking group and the non-smoking group samples was compared. Pivotal genes and pathways responsible for key events in epigenetic changes regulated by microRNA binding and DNA methylation have been identified in the present study.

107 up-regulated genes have been screened out via the strategy of overlapping DEGs and DEM target genes, as plotted in Figure 2E. The molecular function enriched was mainly relevant to ubiquitination, indicating that the regulation of ubiquitin may play an important role in the pathogenesis caused by smoking. KEGG analysis revealed enriched pathways including amyotrophic lateral sclerosis, TGF-beta signaling pathway, autophagy, mTOR signaling pathway, and EGFR tyrosine kinase inhibitor resistance pathway, as shown in Figure 3A. Transforming growth factor-β (transforming growth factor-β, TGF-β) is a multi-functional cytokine that plays a dual role of anti-inflammatory and pro-inflammatory, and can act on the body’s immune response, inflammation, tissue repair, and embryonic development (Grunwald et al., 2021; Kim and Christian, 2021; Qiu et al., 2021; Wang et al., 2021; Xu et al., 2021). Studies have found that TGF-β is involved in the development of periodontal inflammation. Smoking promotes the development of chronic periodontitis by increasing the expression levels of IL-17 and TGF-β (C. Wang et al., 2021; Woods et al., 2021; Xu et al., 2021). Mechanistic Target of Rapamycin (mTOR) is an atypical serine/threonine-protein kinase, belonging to the phosphoinositide 3-kinase (PI3K)- related kinase family. The mTOR signal pathway can be activated by various external factors such as growth factors, stress, oxygen, and amino acid, and is involved in many physiological processes, such as liposome synthesis, energy metabolism, and autophagy. The inducible knockout of mTOR in mouse airway epithelial cells and alveolar type II epithelial cells will exacerbate smoking-induced inflammation, autophagy, necrosis, and apoptosis, which will lead to airway inflammation and emphysema (Liu et al., 2021; Wu et al., 2021; Ye et al., 2021). MircoRNA-mRNA regulatory network in Figure 3C demonstrated that KPNA6 and TOR1AIP2 were targeted by four miRNAs. SMAD4, BCL2L11 were targeted by three miRNA. Smad4 is a key molecule in the TGFbeta pathway. The phosphorylated Smad2 and Smad3 binding with Smad4 can form a molecular compound to achieve TGF-β1 signal transduction into the cell nucleus, thus initiating the biological effect (Dai et al., 2021; Zhang and Qiu, 2021). However, the functions of the other three genes in smoking-related pathological changes are poorly understood.

A total of 65 lower-expressed genes were filtered by taking the intersection of DEGs and DEM target genes, as illustrated in Figure 2F. It was found via enrichment that proteins encoded were mostly expressed around nerve synapses and mainly involved in biological processes related to catabolism and apoptosis. P53 signaling pathway, prostate cancer, and viral carcinogenesis pathway enriched by KEGG database suggested that these genes may play an important part in the pathogenesis of smoking-related cancers, as shown in Figure 3B. Figure 3D demonstrated that more than eight genes were targeted by hsa-miR-16-5p, hsa-miR-6127, hsa-miR-6124, and hsa-miR-25-3p, respectively. Sachin Kumar et al. found that hsa-miR-25-3p was significantly down-regulated in the serum of patients with non-small cell lung cancer, and the expression was significantly correlated with the stage of cancer (Kumar et al., 2020). He et al. found that hsa-miR-16 can inhibit the proliferation of nasopharyngeal carcinoma cells by inhibiting the activation of PI3K/AKT and MAPK signaling pathways (He and Huang, 2017). Therefore, the down-regulation of miR-16 in smokers may be related to carcinogenesis caused by smoking. However, the role of hsa-miR-6127 and hsa-miR-6124 play in cigarette smoking-related pathology has not been reported.

As we all know, lncRNA and cirRNA, as epigenetic regulatory molecules, are widely found in the regulation of gene expression (Gu et al., 2021; Li et al., 2021). miRNA induces gene silencing and down-regulates gene expression by binding to mRNA. However, the upstream molecules circRNA and lncRNA can influence the binding of miRNA to downstream target gene mRNA by competing for binding with miRNA response elements, thereby up-regulating downstream target gene expression. This interaction is called the ceRNA network (Li et al., 2021). It was reported that CSE inhibits insulin production by upregulating TXNIP via MALAT1-mediated downregulation of miR-17 causing reduced β-cells function (Sun et al., 2018). Ma et al. found that cigarette smoke extract (CSE) caused elevated epithelial-mesenchymal transition (EMT) and the increases of circ0061052 in HBE cells. By binding miR-515-5p competitively to regulate the expression of FoxC1/Snail, circ0061052 was involved in the epithelial-mesenchymal transition of epithelial cells during cigarette smoke-induced airway remodeling (Ma et al., 2020). Luo et al. (2020) reported the role of long noncoding RNA MEG3/miR-378/GRB2 axis in neuronal autophagy and neurological functional impairment in ischemic stroke. GRB2 is one of the hub genes in our study and its binding to miR-378a-3p has been predicted, as illustrated in Figure 6D. As a result, ceRNA regulatory network of GRB2 may also play an important part in CSE induced pathogensis. In addition, the ceRNA networks of hub genes were predicted and constructed providing research clues for further exploration, as plotted in Figures 3E, 6D‒E.

It was showed that 324 up-regulated genes with DNA hypomethylation were mainly enriched in neurodegeneration, amyotrophic lateral sclerosis, Alzheimer’s disease, and Huntington disease in terms of the KEGG pathway, as shown in Figure 4A. Therefore, these genes may play an important role in the neurological pathogenesis caused by cigarette smoking. It has been frequently reported that smoking may increase the risk of various neuropsychiatric diseases, such as cognitive disorder, psychopathy, depression, and anxiety. Inflammation and oxidative stress may play a crucial role in the pathogenesis of smoking-induced nervous injury (Liu et al., 2020; Na et al., 2014). After the application of the MOCDE plugin, 10 hub genes have been screened out, among which UBE2N is the gene with the highest degree of connectivity. UBE2N gene which encodes the E2 ubiquitin-conjugating enzyme responsible for the repair of DNA after replication plays an important role in ubiquitin-mediated protein degradation. Modification of proteins with ubiquitin is an important cellular mechanism for targeting abnormal or short-lived proteins for degradation (Dikshit et al., 2018; Zhu Q. et al., 2020). The up-regulation of UBE2N may be regarded as a defense mechanism against the smoking-induced abnormality of protein.

There were 204 low expressed genes with hypermethylation. GO and KEGG enrichment analysis suggested that they were mainly enriched in cardiovascular system functions, including adrenergic signaling in cardiomyocytes, platelet activation, and regulation of actin filament-based process, as shown in Figure 4C. Platelet activation is commonly seen in the blood of smokers. Platelets isolated from smokers exhibit spontaneous aggregation (Nardin et al., 2020). After exposure to smoker serum, platelets isolated from non-smokers exhibit a high degree of aggregation (Pamukcu et al., 2011). Smoking may reduce the availability of platelet-derived NO and reduce the sensitivity of platelets to exogenous NO, leading to increased activation and adhesion (Korniluk et al., 2019). Smoking can also lead to impaired vasodilation. Several studies have shown that both active and passive smoking are related to decreased vasodilation. For humans, exposure to cigarette smoke impairs endothelial dependence vasodilation (EDV) in the large vascular bed (such as coronary and brachial arteries) and the microvascular bed (Tousoulis et al., 2012). The enrichment results indicated that the Adrenergic signaling pathway may play a part in smoking-induced vascular spasm.

It is worth noting that miRNA and DNA methylation may synergistically regulate the abnormal expression of certain genes in smokers. Fourty three genes such as GRB2, UBE2N, TRMT2A, AMBRA1, CAMKK1, and ZNF516 showed enhanced expression accompanied by DNA hypomethylation and reduced miRNA expression. Similarly, 14 genes including FBXL20, CLN8, PDZD8, and NAV1 were down-regulated with the same dual regulation pattern. KEGG enrichment analysis showed that these genes were involved in the EGFR signaling pathway, insulin pathway, focal adhesion kinase-mediated signaling events, mTOR signaling pathway, and internalization of ErbB1, as plotted in Figure 5A. EEF2 and CNBP were highly expressed in the hematopoietic system and vasculature, as shown in Figure 5C. Among the down-regulated genes plotted in Figure 5D, GFRA1 showed low expression in the hematopoietic system and vasculature. It is suggested that these three genes may play an important role in the changes of blood vessels and the hematopoietic system (including monocyte and osteoclastogenesis) caused by cigarette smoking.

Since there are no explicit drugs to improve or reverse the pathophysiological conditions caused by smoking, online databases were used to help predict some drugs. At present, CMap is a useful tool for exploring new drugs and reusing existing drugs. Its effectiveness has been confirmed by many studies (Gao et al., 2019). From the CMap database, 10 chemical substances were identified, including Gly-His-Lys, wortmannin, tetracaine, diethylstilbestrol, and calcium pantothenate, as listed in Table 1. They may have significant potential therapeutic effects for smoking-caused pathological conditions. Many studies have proved the important role of peptidergic mechanisms in regulating various body functions. Gly-His-Lys (Bobyntsev et al., 2015; Sever’yanova Lcapital and Dolgintsev, 2017) reveals the versatility of this effect. It has a regulatory effect on cell growth and differentiation, promotes wound healing by stimulating fibroblasts to synthesize collagen, promotes liver repair and liver cell regeneration, stimulates hair growth, and has an anti-inflammatory effect on damaged tissues. It can also act on the nervous system and function as an anti-anxiety drug (Lassila et al., 1989; Kukowska and Dzierzbicka, 2014; Kukowska et al., 2015).

After topological structure analysis of protein interaction of all overlapping dual-regulated genes, the top 4 pivotal genes turned out to be HSPA4, GRB2, PRKCA, and BCL2L1. The BCL2L1 encoded protein belongs to the BCL-2 protein family and acts as an anti- or pro-apoptotic regulator involved in a variety of cellular activities (Dai et al., 2019). It is located in the outer mitochondrial membrane and has been shown to regulate the opening of the outer mitochondrial membrane channel (VDAC) which plays an important role in apoptosis. The protein encoded by GRB2 binds epidermal growth factor receptor and contains one SH2 domain and two SH3 domains. Its two SH3 domains take part in the formation of complexes with the proline-rich regions of other proteins, and its SH2 domain binds to tyrosine phosphorylation sequences and participates in signal transduction pathways (Tari and Lopez-Berestein, 2001). The protein kinase C (PKC) encoded by the PRKCA gene is a family of serine and threonine-specific protein kinases that can be activated by calcium and second messenger diglycerides (Huang et al., 2020). Members of the PKC family phosphorylate multiple protein targets and are known to participate in multiple cell signaling pathways. It has been reported that this kinase plays a role in many different cellular processes, such as cell adhesion, cell transformation, cell cycle checkpoints, and cell volume control. Knockout studies in mice indicate that this kinase may be the basic regulator of myocardial contractility and Ca(2+) influx in cardiomyocytes (Li et al., 2017). Heat shock proteins promote cell survival by supporting protein folding, stabilization and the unfolding of denatured proteins. Heat shock protein 70 (HSP70) is a 70kd stress-inducing protein whose intracellular expression can increase the tolerance of cardiomyocytes to ischemia/reperfusion injury (Fernandez-Fernandez and Valpuesta, 2018). It also has been reported that extracellular HSP70 is a modulator of inflammatory response. The enhanced expression of HSPA4 encoding HSP70 was demonstrated in the pressure zone of experimental tooth movement, indicating that the intracellular defense system was activated to ensure cell survival (Fontaine et al., 2016; Zhang et al., 2018).

The study revealed a series of deferentially expressed genes that are associated with epigenetic variations in miRNA expression and DNA methylation in smokers. By overlapping DEGs and DEM-targeted genes, 107 low miRNA-targeted up-regulated genes and 65 high-miRNA down-regulated genes were obtained, which were enriched in autophagy signaling pathways and protein ubiquitination pathways, respectively. In addition, 324 low-methylation-high-expression genes and 204 high-methylation-low-expression genes are respectively related to the degeneration of the nervous system and the function of the cardiovascular system. Interestingly, 43 genes were up-regulated under the dual regulation of reduced miRNA and hypomethylation, while 14 genes were down-regulated under the dual regulation of increased miRNA and hypermethylation. Ten chemicals have been identified as putative therapeutic agents for pathological conditions caused by smoking. In addition, HSPA4, GRB2, PRKCA, and BCL2L1 were regarded as hub genes and could play a fundamental role in pathological conditions caused by smoking and may be used as the biomarkers for precise diagnosis and targets for future therapies of smoking-related diseases.

## Data Availability

The datasets presented in this study can be found in online repositories. The names of the repository/repositories and accession number(s) can be found in the article/Supplementary Material.
