# Supplementary material for: Epigenetic Biomarkers Screening of Non-Coding RNA and DNA Methylation Based on Peripheral Blood Monocytes in Smokers
Source: Front Genet. 2022 Feb 11;13:766553. doi: 10.3389/fgene.2022.766553 (PMC8882369; doi:10.3389/fgene.2022.766553)
Supplement: Supplementary file 1 [file DataSheet1.docx]


Supplementary Table 1. Gene ontology and KEGG pathway analysis of DEGs associated with aberrant DNA methylation between smokers and healthy non-smokers samples.

| **Category** | **Term** | **Count** | **Precent (%)** | **P value** |
| --- | --- | --- | --- | --- |
| ***Low miRNA targeting up-regulated genes*** | |  |  |  |
| Biological Process | covalent chromatin modification | 9 | 0.09 | 0.000824104 |
| Biological Process | in utero embryonic development | 8 | 0.08 | 0.000439111 |
| Biological Process | transmembrane receptor protein serine/threonine kinase signaling pathway | 8 | 0.08 | 0.000679203 |
| Biological Process | histone modification | 8 | 0.08 | 0.002802611 |
| Biological Process | proteasomal protein catabolic process | 8 | 0.08 | 0.00441299 |
| Cellular Component | nuclear envelope | 7 | 0.07 | 0.010155007 |
| Cellular Component | transferase complex, transferring phosphorus-containing groups | 5 | 0.05 | 0.010164871 |
| Cellular Component | spindle | 5 | 0.05 | 0.047899275 |
| Cellular Component | fibrillar center | 4 | 0.04 | 0.005899474 |
| Cellular Component | mitotic spindle | 4 | 0.04 | 0.008995038 |
| Molecular Function | ubiquitin protein ligase binding | 6 | 0.06 | 0.005643901 |
| Molecular Function | ubiquitin-like protein ligase binding | 6 | 0.06 | 0.007596257 |
| Molecular Function | ubiquitin-protein transferase activity | 6 | 0.06 | 0.031284672 |
| Molecular Function | protein serine/threonine kinase activity | 6 | 0.06 | 0.031590536 |
| Molecular Function | ubiquitin-like protein transferase activity | 6 | 0.06 | 0.038821209 |
| KEGG Pathway | Amyotrophic lateral sclerosis | 7 | 0.11 | 0.024215761 |
| KEGG Pathway | TGF-beta signaling pathway | 5 | 0.08 | 0.000836412 |
| KEGG Pathway | Autophagy - animal | 5 | 0.08 | 0.00496213 |
| KEGG Pathway | mTOR signaling pathway | 5 | 0.08 | 0.007371327 |
| ***High miRNA targeting down-regulated genes*** | |  |  |  |
| Biological Process | protein polyubiquitination | 7 | 0.12 | 9.36E-05 |
| Biological Process | regulation of cell cycle phase transition | 7 | 0.12 | 0.000777657 |
| Biological Process | protein targeting | 6 | 0.1 | 0.002721612 |
| Biological Process | positive regulation of catabolic process | 6 | 0.1 | 0.003007562 |
| Biological Process | regulation of intrinsic apoptotic signaling pathway | 5 | 0.08 | 0.000154641 |
| Cellular Component | cell projection membrane | 5 | 0.08 | 0.004017706 |
| Cellular Component | integral component of postsynaptic membrane | 4 | 0.07 | 0.000270478 |
| Cellular Component | intrinsic component of postsynaptic membrane | 4 | 0.07 | 0.000325918 |
| Cellular Component | integral component of synaptic membrane | 4 | 0.07 | 0.000689493 |
| Cellular Component | intrinsic component of synaptic membrane | 4 | 0.07 | 0.000963563 |
| Molecular Function | DNA-binding transcription activator activity, RNA polymerase II-specific | 7 | 0.11 | 0.000648595 |
| Molecular Function | DNA-binding transcription activator activity | 7 | 0.11 | 0.000683911 |
| Molecular Function | actin binding | 6 | 0.1 | 0.003324432 |
| Molecular Function | secondary active transmembrane transporter activity | 4 | 0.07 | 0.00821799 |
| Molecular Function | ubiquitin protein ligase binding | 4 | 0.07 | 0.016342544 |
| KEGG Pathway | Endocytosis | 5 | 0.15 | 0.003243448 |
| KEGG Pathway | p53 signaling pathway | 3 | 0.09 | 0.003147477 |
| KEGG Pathway | Prostate cancer | 3 | 0.09 | 0.006983351 |
| KEGG Pathway | Ubiquitin mediated proteolysis | 3 | 0.09 | 0.018836095 |
| KEGG Pathway | Viral carcinogenesis | 3 | 0.09 | 0.049263642 |

Supplementary Table 2. Gene ontology and KEGG pathway analysis of DEGs associated with aberrant DNA methylation between smokers and healthy non-smokers samples.

| **Category** | **Term** | **Count** | **Precent (%)** | ***P* value** |
| --- | --- | --- | --- | --- |
| ***Hypomethylation and up-regulated genes*** | | | | |
| Biological Process | ribonucleoprotein complex biogenesis | 22 | 7.51 | 5.23E-06 |
| Biological Process | ncRNA metabolic process | 18 | 6.14 | 0.000726912 |
| Biological Process | ribosome biogenesis | 16 | 5.46 | 2.66E-05 |
| Biological Process | anatomical structure homeostasis | 15 | 5.12 | 0.006462599 |
| Biological Process | neutrophil degranulation | 15 | 5.12 | 0.009154299 |
| Cellular Component | mitochondrial inner membrane | 20 | 6.67 | 8.36E-05 |
| Cellular Component | mitochondrial matrix | 19 | 6.33 | 0.000154786 |
| Cellular Component | chromosomal region | 16 | 5.33 | 9.43E-05 |
| Cellular Component | vacuolar membrane | 12 | 4.00 | 0.035227832 |
| Cellular Component | secretory granule lumen | 11 | 3.67 | 0.011439761 |
| Molecular Function | ubiquitin-like protein ligase binding | 14 | 4.62 | 0.000727586 |
| Molecular Function | catalytic activity, acting on RNA | 14 | 4.62 | 0.005142478 |
| Molecular Function | ubiquitin protein ligase binding | 13 | 4.29 | 0.001235971 |
| Molecular Function | phosphatase binding | 10 | 3.30 | 0.001432577 |
| Molecular Function | cadherin binding | 10 | 3.30 | 0.04992678 |
| KEGG Pathway | Pathways of neurodegeneration - multiple diseases | 16 | 11.27 | 0.008885383 |
| KEGG Pathway | Amyotrophic lateral sclerosis | 13 | 9.15 | 0.01146189 |
| KEGG Pathway | Alzheimer disease | 12 | 8.45 | 0.028134935 |
| KEGG Pathway | Huntington disease | 11 | 7.75 | 0.018295831 |
| KEGG Pathway | Hepatitis B | 9 | 6.34 | 0.002103332 |
| ***Hypermethylation and down-regulated genes*** | | | | |
| Biological Process | regulation of anatomical structure size | 15 | 7.73 | 0.000185067 |
| Biological Process | dephosphorylation | 14 | 7.22 | 0.000579296 |
| Biological Process | aging | 12 | 6.19 | 7.63E-05 |
| Biological Process | regulation of actin filament-based process | 12 | 6.19 | 0.000724748 |
| Biological Process | protein dephosphorylation | 11 | 5.67 | 0.000563829 |
| Cellular Component | focal adhesion | 12 | 6.12 | 0.001054984 |
| Cellular Component | cell-substrate junction | 12 | 6.12 | 0.001216245 |
| Cellular Component | postsynaptic specialization | 11 | 5.61 | 0.000567169 |
| Cellular Component | early endosome | 11 | 5.61 | 0.001581826 |
| Cellular Component | postsynaptic density | 10 | 5.10 | 0.001291486 |
| Molecular Function | actin binding | 14 | 7.07 | 0.000308676 |
| Molecular Function | protein serine/threonine kinase activity | 11 | 5.56 | 0.007201697 |
| Molecular Function | ubiquitin-protein transferase activity | 10 | 5.05 | 0.018429744 |
| Molecular Function | DNA-binding transcription activator activity, RNA polymerase II-specific | 10 | 5.05 | 0.022437631 |
| Molecular Function | DNA-binding transcription activator activity | 10 | 5.05 | 0.023690978 |
| KEGG Pathway | Epstein-Barr virus infection | 7 | 7.07 | 0.011581323 |
| KEGG Pathway | Rap1 signaling pathway | 6 | 6.06 | 0.04313712 |
| KEGG Pathway | Platelet activation | 5 | 5.05 | 0.01762052 |
| KEGG Pathway | Adrenergic signaling in cardiomyocytes | 5 | 5.05 | 0.036232638 |
| KEGG Pathway | Adherens junction | 4 | 4.04 | 0.010883227 |

Supplementary Table 3. Hub genes with the top 10 degrees of both high expression with hypomethylation and low expression genes with hypermethylation.

| **Gene** | **Gene Description** | **Degree** |
| --- | --- | --- |
| ***Hypomethylation and High-Expression hub Genes*** | |  |
| UBE2N | ubiquitin conjugating enzyme E2 N | 28 |
| HSPA4 | heat shock protein family A (Hsp70) member 4 | 27 |
| HSPA9 | heat shock protein family A (Hsp70) member 9 | 23 |
| HSPD1 | heat shock protein family D (Hsp60) member 1 | 22 |
| EEF2 | eukaryotic translation elongation factor 2 | 21 |
| ATP5A1 | ATP synthase, H+ transporting, mitochondrial F1 complex, alpha subunit 1 | 19 |
| CCT4 | chaperonin containing TCP1 subunit 4 | 19 |
| PSMC2 | proteasome 26S subunit, ATPase 2 | 19 |
| CCT8 | chaperonin containing TCP1 subunit 8 | 17 |
| MRPL4 | mitochondrial ribosomal protein L4 | 17 |
| ***Hypermethylation and Low-Expression hub Genes*** | |  |
| BTRC | beta-transducin repeat containing E3 ubiquitin protein ligase | 14 |
| FBXL20 | F-box and leucine rich repeat protein 20 | 11 |
| UBE3A | ubiquitin protein ligase E3A | 10 |
| HECTD1 | HECT domain E3 ubiquitin protein ligase 1 | 9 |
| MEX3C | mex-3 RNA binding family member C | 9 |
| RNF213 | ring finger protein 213 | 9 |
| UBE3C | ubiquitin protein ligase E3C | 9 |
| ANAPC5 | anaphase promoting complex subunit 5 | 8 |
| LONRF1 | LON peptidase N-terminal domain and ring finger 1 | 8 |
| BCL2L1 | BCL2 like 1 | 7 |

Supplementary Table 4. DEGs associated with both specific miRNA and DNA methylation CpG sites between GDM and healthy samples.

| **Gene** | **DMP chromosome location miRNA** | | | |
| --- | --- | --- | --- | --- |
| ***Up-regulated genes affected by both low miRNA and hypomethylation*** | | | | |
| AGPAT3 | cg10636297 | chr21:45402484-45402842 | 3'UTR | hsa-miR-744-5p |
| ALDH6A1 | cg01906922 | chr14:74551326-74551706 | TSS1500 | hsa-miR-4284 |
| AMBRA1 | cg03364891 | chr11:46410921-46414687 | 3'UTR | hsa-miR-331-3p |
| ATPAF1 | cg04138976 | chr1:47133674-47134395 | TSS1500 | hsa-miR-151a-5p |
| BCL2L11 | cg07547695 | chr2:111875206-111880965 | 5'UTR | hsa-miR-221-3p |
| CAMKK1 | cg13379195 | chr17:3795519-3796868 | 5'UTR | hsa-miR-221-3p |
| CNBP | cg02564756 | chr3:128902134-128902978 | TSS200 | hsa-miR-197-3p |
| CNBP | cg04793090 | chr3:128902134-128902978 | TSS1500 | hsa-miR-26a-5p |
| CRK | cg05348421 | chr17:1358888-1359814 | TSS1500 | hsa-miR-4284 |
| CRK | cg11280964 | chr17:1358888-1359814 | TSS1500 | hsa-miR-4463 |
| CSGALNACT1 | cg03649589 | chr8:19459905-19460137 | TSS1500 | hsa-miR-199a-5p |
| CSGALNACT1 | cg23904224 | chr8:19459905-19460137 | TSS1500 |  |
| CSGALNACT1 | cg24280945 | chr8:19459905-19460137 | TSS1500 |  |
| DCTN5 | cg05002041 | chr16:23652387-23652918 | 5'UTR | hsa-miR-18b-5p |
| DCTN5 | cg08350734 | chr16:23652387-23652918 | TSS1500 |  |
| DCTN5 | cg09500317 | chr16:23652387-23652918 | TSS1500 |  |
| DPP8 | cg04799823 | chr15:65809685-65810168 | TSS200 | hsa-miR-197-3p |
| DPP8 | cg18202577 | chr15:65809685-65810168 | 5'UTR | hsa-miR-483-5p |
| DPP8 | cg25130390 | chr15:65809685-65810168 | TSS200 |  |
| DPP8 | cg25457200 | chr15:65809685-65810168 | TSS1500 |  |
| DPP8 | cg27056853 | chr15:65809685-65810168 | 5'UTR |  |
| ECD | cg24044147 | chr10:74927642-74928063 | TSS1500 | hsa-miR-361-5p |
| EEF2 | cg13634151 | chr19:3984962-3985722 | TSS1500 | hsa-miR-4701-3p |
| FXR1 | cg01816191 | chr3:180630152-180630996 | TSS1500 | hsa-miR-4463 |
| GABPB1 | cg14821257 | chr15:50646437-50647742 | 5'UTR | hsa-let-7d-5p |
| GRB2 | cg04849856 | chr17:73401217-73401988 | 5'UTR | hsa-miR-378a-3p |
| GRB2 | cg18385501 | chr17:73401217-73401988 | TSS200 |  |
| HSPA4 | cg13778073 | chr5:132387100-132388369 | 1stExon | hsa-miR-197-3p |
| ING1 | cg00743107 | chr13:111364718-111368530 | 5'UTR | hsa-miR-4284 |
| KAT2A | cg11592497 | chr17:40274523-40275360 | TSS200 | hsa-miR-331-3p |
| KIAA1958 | cg03345938 | chr9:115248728-115249744 | TSS1500 | hsa-miR-197-3p |
| LDHA | cg01316516 | chr11:18415922-18416680 | 5'UTR | hsa-miR-324-5p |
| LDHA | cg11166108 | chr11:18417667-18417925 | 5'UTR | hsa-miR-4284 |
| LGALS8 | cg04027302 | chr1:236687071-236687608 | TSS1500 | hsa-miR-127-3p |
| MKRN1 | cg06784339 | chr7:140178289-140179467 | 1stExon | hsa-miR-574-3p |
| NUDT21 | cg02941219 | chr16:56484865-56485774 | 1stExon | hsa-miR-27b-3p |
| PAFAH1B2 | cg10398005 | chr11:117014699-117015560 | TSS200 | hsa-miR-4284 |
| PAICS | cg27247723 | chr4:57301340-57302670 | 5'UTR | hsa-miR-371b-5p |
| PATZ1 | cg13003054 | chr22:31741045-31743707 | 1stExon | hsa-miR-326 |
| PATZ1 | cg14957214 | chr22:31741045-31743707 | TSS1500 |  |
| PDPK1 | cg14444710 | chr16:2588034-2588556 | TSS1500 | hsa-miR-4725-5p |
| PER2 | cg08202720 | chr2:239196923-239197900 | 5'UTR | hsa-miR-30a-5p |
| PER2 | cg11903188 | chr2:239196923-239197900 | 5'UTR |  |
| PPARGC1B | cg18547262 | chr5:149109570-149111750 | TSS200 | hsa-miR-361-3p |
| PRKCA | cg09645572 | chr17:64298047-64299617 | TSS1500 | hsa-miR-371b-5p |
| RBM33 | cg01115569 | chr7:155436673-155437817 | TSS1500 | hsa-miR-221-3p |
| RBM33 | cg01807375 | chr7:155573602-155573826 | 3'UTR |  |
| RBM33 | cg14615491 | chr7:155436673-155437817 | TSS1500 |  |
| RRAGD | cg22264975 | chr6:90120874-90122370 | 1stExon | hsa-miR-130b-3p |
| SOCS6 | cg13738652 | chr18:67955692-67957062 | TSS1500 | hsa-miR-27b-3p |
| SPATA2 | cg01458420 | chr20:48531603-48532316 | TSS1500 | hsa-miR-130b-3p |
| SPATA2 | cg11652597 | chr20:48522602-48523134 | 3'UTR |  |
| SPATA2 | cg19221489 | chr20:48531603-48532316 | TSS1500 |  |
| TIAM1 | cg07462053 | chr21:32716044-32716485 | 5'UTR | hsa-miR-221-3p |
| TIAM1 | cg25046584 | chr21:32929927-32932017 | TSS1500 |  |
| TLE3 | cg08342270 | chr15:70387929-70393206 | 1stExon | hsa-miR-222-3p |
| TRMT2A | cg10614909 | chr22:20104374-20105729 | TSS1500 | hsa-miR-125a-5p |
| TRMT2A | cg22126499 | chr22:20103648-20103923 | 3'UTR |  |
| TRMT5 | cg26161780 | chr14:61447663-61448141 | TSS1500 | hsa-miR-222-3p |
| TRPS1 | cg05017276 | chr8:116681380-116681623 | TSS200 | hsa-miR-125a-5p |
| TRPS1 | cg12569516 | chr8:116681380-116681623 | 1stExon | hsa-miR-222-3p |
| UBE2N | cg15930798 | chr12:93834935-93836089 | TSS200 | hsa-miR-744-5p |
| UEVLD | cg05578937 | chr11:18610010-18610463 | TSS200 | hsa-miR-575 |
| ZNF516 | cg13049398 | chr18:74153239-74155073 | 5'UTR | hsa-miR-575 |
| ZNF516 | cg15491120 | chr18:74153239-74155073 | 5'UTR |  |
| ZNF516 | cg20132820 | chr18:74170530-74170791 | 5'UTR |  |
| ***Down-regulated genes affected by both high miRNA and hypermethylation*** | | | | |
| UBE3C | cg01939428 | chr7:156931422-156932075 | TSS1500 | hsa-miR-16-5p |
| TSC22D2 | cg14056942 | chr3:150125972-150128689 | 1stExon | hsa-miR-16-5p |
| SLC6A4 | cg26126367 | chr17:28562387-28563186 | 5'UTR | hsa-miR-16-5p |
| NAV1 | cg01058902 | chr1:201708787-201709429 | TSS1500 | hsa-miR-6127 |
| NAV1 | cg09795973 | chr1:201798213-201798698 | 3'UTR |  |
| NAV1 | cg23801168 | chr1:201617041-201619788 | TSS1500 |  |
| KCND3 | cg04843968 | chr1:112524468-112525385 | 5'UTR | hsa-miR-6124 |
| KCND3 | cg17898124 | chr1:112531559-112533773 | 5'UTR |  |
| KCND3 | cg22040158 | chr1:112524468-112525385 | 5'UTR |  |
| KCND3 | cg22482502 | chr1:112524468-112525385 | 5'UTR |  |
| GLYR1 | cg01565912 | chr16:4896606-4898243 | TSS1500 | hsa-miR-6127 |
| FBXL20 | cg10589848 | chr17:37557366-37558982 | TSS1500 | hsa-miR-16-5p |
| CLN8 | cg10355466 | chr8:1733267-1733929 | 3'UTR | hsa-miR-25-3p |
| CLN8 | cg13996619 | chr8:1711234-1712654 | 5'UTR |  |
| CLN8 | cg23952859 | chr8:1711234-1712654 | 5'UTR |  |

Supplementary Table 5. The gene list of dual regulated genes. Left: up regulated genes with hypomethlyation and lower-expressed miRNA.

Right: down regulated genes with hypermethlyation and upper-expressed miRNA.

| *Up regulated* | *Down regulated* |
| --- | --- |
| GRB2 | FBXL20 |
| UBE2N | CLN8 |
| TRMT2A | PDZD8 |
| AMBRA1 | NAV1 |
| CAMKK1 | GLYR1 |
| ZNF516 | KCND3 |
| LDHA | MYBL1 |
| KAT2A | TSC22D2 |
| FXR1 | TGFBR3 |
| EEF2 | GFRA1 |
| PATZ1 | SLC6A4 |
| RRAGD | FMNL3 |
| TRPS1 | BCL2L1 |
| CNBP | UBE3C |
| ALDH6A1 |  |
| SPATA2 |  |
| PPARGC1B |  |
| NUDT21 |  |
| TRMT5 |  |
| SOCS6 |  |
| AGPAT3 |  |
| HSPA4 |  |
| PDPK1 |  |
| PER2 |  |
| GABPB1 |  |
| BCL2L11 |  |
| CRK |  |
| ING1 |  |
| PRKCA |  |
| TLE3 |  |
| RBM33 |  |
| DPP8 |  |
| PAICS |  |
| ECD |  |
| TIAM1 |  |
| CSGALNACT1 |  |
| MKRN1 |  |
| KIAA1958 |  |
| ATPAF1 |  |
| PAFAH1B2 |  |
| LGALS8 |  |
| DCTN5 |  |
| UEVLD |  |
